# Potential of dredged bioremediated marine sediment for strawberry cultivation

**DOI:** 10.1038/s41598-020-76714-x

**Published:** 2020-11-16

**Authors:** Juan José Martínez-Nicolás, Pilar Legua, Dámaris Núñez-Gómez, Rafael Martínez-Font, Francisca Hernández, Edgardo Giordani, Pablo Melgarejo

**Affiliations:** 1 Department of Plant Science and Microbiology, Escuela Politécnica Superior de Orihuela (EPSO), Universidad Miguel Hernández de Elche (UMH), Ctra. Beniel, Km 3.2, 03312 Orihuela, Alicante Spain; 2 Fruit and Vegetable Department, University of Florence, Viale Delle Idee, 30, 50019 Sesto Fiorentino, Firenze Italy

**Keywords:** Agroecology, Plant sciences, Environmental sciences

## Abstract

For the maintenance of the economic activity of the ports, it is necessary to dredge the marine sediments in order to guarantee their depth. These sediments, considered by European legislation as residues, present relevant limitations of use and generate environmental and economic problems concerning their final disposal. In this context, the present work aims to identify the phytoremediated dredged sediments potential as an alternative to the traditional substrate (peat) in horticultural growing through two-years controlled strawberry cultivation. The growing media mixes used were: (1) 100% peat (Pt) as a control substrate; (2) 100% dredged remediated sediment (DRS); (3) 50% each (Pt-DRS). The dredged sediment, plant drainage and strawberry plant parts (leaves, stems, roots, and fruits) were analyzed to mineral elements, heavy metal contents, and pesticide residues (polycyclic aromatic hydrocarbons, polychlorinated biphenyls and specific fumigants) during the experimental period. Only seven (Mn, Fe, Zn, Mo, Al, Mn and Ni) of the twenty-two metals and two (nitrates and fluorene) of the six hundred-thirteen pesticides analyzed were detected in the strawberry fruits. In all the cases, values detected were under the Spanish and European legal limit. The suitability of strawberry fruits for fresh and/or processed consumption with no risk was confirmed. Based on the results, can be affirmed that the dredged remediated sediment can be used as a culture substrate, alone or mixed with other substrates. Additional researches should be carried out to confirm the sediment characteristics and compare with other substrates to improve the physical and chemical properties.

## Introduction

The worldwide importance and economic influence of seaports are indisputable. However, to do this activity it is necessary to periodically dredge the marine sediments to guarantee the safe passage of large-draft boats, allowing preserve both traffic and port competitiveness^1^.

 Being an economical necessary activity, and due to the large volumes of marine sediments generated annually in both European Union seaports (around 200 million m^3^ every year)^2^ and the rest world seaports, in recent years the need to investigate new options and alternative uses of dredged sediments have been identified to minimize the derived environmental impacts^3^. These impacts are mainly focused on: (1) changes in the physical, chemical and biological characteristics of marine and coastal ecosystems due to the movement of sediments^4^; (2) impacts derived from the transport and disposal of sediments in suitable places; and (3) specific requirements for management, as the sediments may contain pollutants from local anthropogenic activities and / or river contributions^5^. This last point has motivated current international legislation to consider dredged sediments as contaminated waste, limiting their use, application and disposal. However, specific studies, such as developed by Buceta et al.^3^ identified the “fine granular size” that corresponds to less than 10% in the investigated sediment, as the part generally contaminated by metals and organic pollutants. Therefore, it can be assumed that in many cases, and for the most part, these sediments could be considered a resource rather than a residue.

Peat has been considered the most used substrate in the last decade due to its physical–chemical characteristics and suitability for many species and cultivation systems^6–8^. Nevertheless, strong demand and unsustainable exploration of peatlands around the world is creating availability problems and signs of depletion. For this reason, it is increasingly necessary to identify and study new substrates that make it possible to reduce, or even replace, the use of peat in agriculture^9–12^. Mattei et al.^13^ demonstrated the viability of phytoremediation dredged sediment as a substrate for plant growth. The term phytoremediation is derived from the Greek word “Phyto” which means plant and “Remedy” which is “equilibrium recovery”, normally, it can be defined as a decontamination process through the use of green plants due to their ability to retain, metabolize and/or accumulate pollutants in water, soil and/or air through accumulation, immobilization, rhizodegradation mechanisms among others^14^. Phytoremediation is considered the most profitable and environmentally friendly soil recovery strategy^15^ that is in continuous development and improvement^16,17^.

In this context, the present work focuses on the study of the phytoremediated dredged sediments potential and suitability as an alternative to the traditional substrate (peat) in horticultural growing media by monitoring the content of mineral elements, metals and pesticide residues in strawberry plant and fruits (*Fragaria x ananassa* Duch.), Variety Monterrey. Thus, the main objective of the research was to provide a sustainable alternative use and environmental appropriate management of dredged marine sediments that are currently considered as a major economic, landscape and environmental problem. Besides, the authors intended that the results of this study contribute to reducing the pressure on natural peatlands, guaranteeing their long-term permanence.

The present work started on the hypothesis that properly treated, dredged marine sediments may cease to be an economic and environmental issue, since they present potential for use as a safe agricultural substrate, and may even be used for other types of uses such as soil regeneration, recovery quarries and/or roads.

## Materials and methods

### Dredged remediated sediments

The dredged remediated sediments (DRS) used in this study came from Leghorn Port (Italy) and were previously phytoremediated for 3 years (**European Project AGRIPORT** - *Agricultural Reuse of Polluted dredged Sediments* - *ECO/08/239,065/S12.53226*). Before starting the experimental assays with strawberry plants, physical (apparent soil density) and chemical (electrical conductivity, pH, cation exchange capacity, total organic C and N, water-soluble and total removable carbon, humic substance fractions, P, Zn, Cd, Ni, Cu, Cr, Pb, Al, Mn, Fe, V, Hg, polychlorobiphenyls and polycyclic aromatic hydrocarbons) analyses were carried out (Table 1). DRS was used in a previous strawberry experiment conducted by the same research group^18^.

**Table 1:** Initial DRS characterization.

Parameter	Value (mean ± standard deviation)
Bulk density (g cm ^−3^)	1.08 ± 0.7
pH	8.1 ± 0.01
C Soluble (mg kg^−1^)	497 ± 0.02
Electrical conductivity (dS m^−1^)	0.30 ± 0.04
Total organic carbon (%)	1.97 ± 0.02
Total extractable carbon (mg kg^−1^)	13.051 ± 520
Humic acid (mg kg^−1^)	8227 ± 158
Fulvic acid (mg kg^−1^)	5542 ± 84
N total (%)	0.13 ± 0.01
NO_3_^-^ (mg kg^−1^)	22.6 ± 1.5
NH_4_^+^ (mg kg^−1^)	0.36 ± 0.05
P_2_O_5_ (%)	0.11 ± 0.02
P total (%)	495 ± 33
Hydrocarbons C > 12 (mg kg^−1^)	207 ± 3.8
Hydrocarbons C < 12 (mg kg^−1^)	< L.Q
PAHs (mg kg^−1^)	49.2 ± 5.3
PCB (mg kg^−1^)	0.039 ± 0.002
Cd_*total*_ (mg kg^−1^)	< L.Q
Mn_*total*_ (mg kg^−1^)	378 ± 28
Mn_*available*_ (mg kg^−1^)	13.8 ± 1.9
Al_*total*_ (mg kg^−1^)	22.744 ± 2319
Al_*available*_ (mg kg^−1^)	3.24 ± 0.13
Fe_*total*_ (mg kg^−1^)	23.073 ± 1676
Fe_*available*_ (mg kg^−1^)	17.8 ± 3.3
V_*total*_ (mg kg^−1^)	30.8 ± 3.7
Hg_*total*_ (mg kg^−1^)	0.075 ± 0.001
Cr _*total*_ (mg kg^−1^)	54.3 ± 1.2
Ni _*total*_ (mg kg^−1^)	34.6 ± 5.33
Cu_*total*_ (mg kg^−1^)	34.3 ± 4.3
Cu_*available*_ (mg kg^−1^)	17.4 ± 2.5
Zn_*total*_ (mg kg^−1^)	248 ± 11
Zn_*available*_ (mg kg^−1^)	37.2 ± 3.3
Pb_*total*_ (mg kg^−1^)	35.2 ± 3.7
Pb_*available*_ (mg kg^−1^)	7.55 ± 0.74

L.Q: Limit of quantification.

In addition, biochemical (dehydrogenase activity, hydrolytic enzyme activities, β-glucosidase, acid phosphatase, protease, phosphodiesterase activity, arylesterase activity, arylsulfatase activity, cellulase activity, protease activity, urease activity and dioxygenase activity) analyses were carried out (data pending publication). The soil toxicity was also evaluated according to the ISO standard method (ISO 11,348–3 1998)^19^.

After phytoremediation, the dredged sediment presented good nutrient content, good biological activity and a low level of contamination, with only a small residual organic contamination. The values complied with the Spanish legislation on substrates for agricultural uses^20^.

### Plant material and experimental design

The experimental trial was carried out during two seasons (2016 and 2017) in an experimental plot of the School of Engineering of Orihuela (Miguel Hernández University) located in Orihuela, SE Spain (38°04′N, 0°58′W, 26 m above sea level).

Five strawberry plants (*Fragaria x ananassa* Duch.), Monterrey cultivar, were planted in rectangular containers (150 cm × 30 cm × 15 cm) covered at the initial grow time with black polythene plastic. Each container was provided with a drainage system to collect excess water and prevent waterlogging. The entire assay was made in triplicate, i.e. three containers (10 plants each) were used for each substrate tested in a randomized complete block design (RCBD). In this sense, dredged remediated sediments and peat mixed in three different proportions were used as substrates: 100% peat (Pt) as a control, 100% dredged remediated sediments (DRS), and 50% each (Pt-DRS). Every year of this study, 90 strawberry plants were used. This same experimental design was used successfully in previous and parallel studies developed by the same research group^18^.

Crop irrigation and nutritional and microelements additives were scheduled according to the methodology developed by the same research group and previously published^18^.

### Plant drainage and plant material characterization

The plant drainages were collected and measured daily. The pH and electrical conductivity were determined with a multiparameter analyzer (CONSORT C860 multiparameter analyzer). For chemical analysis (macro- and microelement concentrations, heavy metals and pesticide residues), aliquots of the drainage were separated in plastic bottles and stored in a refrigerator at 5 °C until used.

At the end of the cultivation, diverse plant parts (roots, stems and leaves) from the different treatments were sampled. All plant samples were analyzed to determine the heavy metal contents (manganese, iron, zinc, copper, molybdenum, lead, cadmium, nickel, chromium, mercury, cobalt, antimony, arsenic, selenium, aluminium, beryllium, tin, strontium, silver, thallium and vanadium) and the pesticide residues as explained below. The results were compared with the specific legal limits both in Europe and Spain.

#### Mineral element and heavy metal determination

The mineral elements were determined in aqueous solution samples. Nitrate, chloride, sulfate, nitrate, bromide and fluoride anions were determined by an ion chromatograph (883 COMPACT IC-PLUS METROHM) equipped with chemical suppression. Nitrates in the plant matrices were determined by water extraction of nitrate anions. A sample exchanger for pH and conductivity measurements (814 USB Sample Processor, METROHM) was used to determine the carbonates and bicarbonates. The results were processed with TIAMO 2.3. METROHM software. Ammoniacal nitrogen was determined by spectrophotometry (VARIAN CARY 50 UV–vis spectrophotometer) following the 4500-NH3 Phenate Method described by Standard Methods for the examination of water and wastewater^21^.

Heavy metal determination was carried out by ICP-MS (mass spectrometry with inductively coupled plasma) using AGILENT 7700-E and AGILENT 7700-X models. For the digestions, MILESTONE ETHOS and MILESTONE ETHOS ONE microwaves were used.

#### Pesticide residue determination

The six hundred and thirteen different pesticides were determined according to the UNE-EN 1562: 2019 method by liquid chromatography with a triple quadrupole mass detector (LC–MS/QqQ, Bruker Evoq Elite) based analysis following acetonitrile (> 99.9% SIGMA ALDRICH) extraction/partition and clean-up by dispersive solid-phase extraction (SPE – OASIS PRIME HLB 3cc/150 mg. WATERS MILFORD). The pesticide residues were identified by retention time comparison with standard calibration solutions (> 99.7% SIGMA ALDRICH) and also by spectral libraries specific to the detected compounds. Besides, polychlorinated biphenyl (PCB) and polycyclic aromatic hydrocarbons (PAH) were analysed according to EPA 1668C 2010 and EPA 8015C 2007 methods, respectively, by gas chromatography with triple quadrupole mass spectrometry detector (GC–MS/QqQ Bruker Scion TQ model). All the pesticide, PAHs and PCBs tests were carried out in triplicate by an accredited Spanish laboratory (KUDAM Laboratory, Alicante).

### Statistical analysis

Statistical analyses were performed using SPSS 24.0 for Windows (SPSS SCIENCE, Chicago, IL, USA). Basic descriptive statistical analysis was followed by an analysis of variance (ANOVA) test for mean comparisons. The method used to discriminate among the means (multiple range test) was Tukey’s test at a 95.0% confidence level. Pearson correlation analyses were also performed.

## Results

### Plant drainage characterization

**Figure 1:**
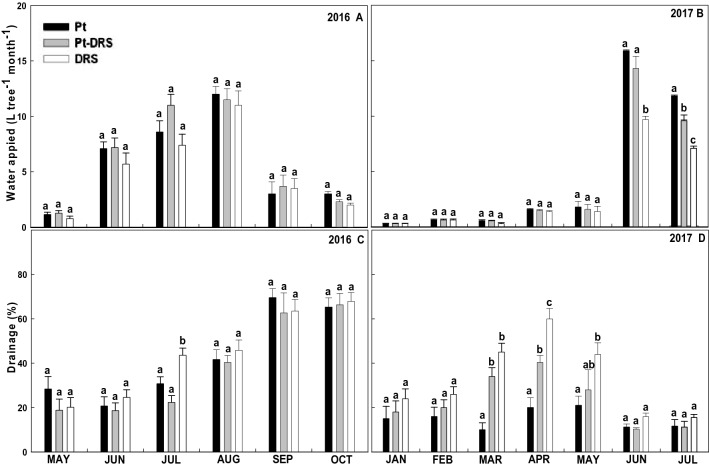
Monthly average of water applied in 2016 (**A**) and 2017 (**B**) and percentage of drainage in 2016 (**C**) and 2017 (**D**) for Monterrey strawberry cultivar. Each bar corresponds to the monthly average relative to each substrate, where Pt corresponds to peat; Pt-DRS 50% mix of peat and dredged sediment; and DRS dredged sediment. Different letters on top of the bars indicate significant differences according to Tukey's HSD test (*p* < 0.05).

The strawberry plants were transplanted to the cultivation benches in May 2016. From this date until May 2017, the amount of water applied to the three substrates (Pt, Pt-DRS, and DRS) was kept essentially the same, but during the summer, the months during the year with greater evapotranspiration, more water was applied to Pt, followed by Pt-DRS and DRS. This difference may be due to the different textures, and therefore, different water retention capacities, of the substrates (Fig. 1A,B). Concerning the drainage volume (Fig. 1C,D), in general, no significant differences in drainage (%) were observed among the different substrates/months, except for the Pt-DRS drainage between March and May 2017, which was significantly higher (Fig. 1D). In volume terms, a constant tendency was identified with some specific variations, so in September and October 2016, drainages close to 60% were reached, while in 2017, the drainage percentage was approximately 20–30% for all substrates except Pt-DRS. This temporary increase in drained volume could have caused washing and dilution of salts and, therefore, a decrease in water drainage electrical conductivity.

#### Electrical conductivity and pH

**Figure 2:**
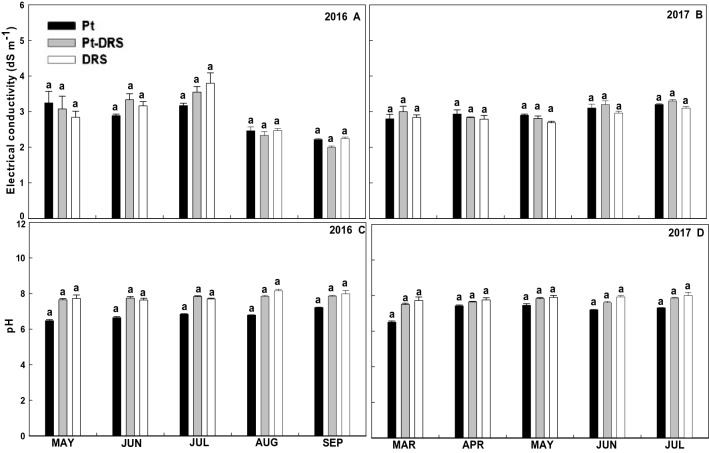
Monthly average electrical conductivity in 2016 (**A**) and 2017 (**B**) and pH in 2016 (**C**) and 2017 (**D**) for Monterrey strawberry cultivar. Each bar corresponds to the monthly average relative to each substrate, where Pt corresponds to peat; Pt-DRS 50% peat and dredged sediment mix; and DRS dredged sediment. Different letters on top of the bars indicate significant differences according to Tukey's HSD test (*p* < 0.05).

The average monthly variations in plant drainage electrical conductivity (EC) and pH are shown in Fig. 2. Generally, the EC values were higher in 2016 than in 2017, reaching values above 4 dS m^−1^ (June 2016). In 2017, EC values close to 3 dS m^−1^ were maintained. On the other hand, in 2016, the Pt pH showed values slightly lower than those in the other two treatments (approximately 8), but in 2017, the average pH values of the three treatments were equalized at approximately 8, and no significant differences were observed (Fig. 2D). This was possibly due to progressive alkalization of the peat over the 2 years of the trial.

#### Macro- and microelements

 The macro- and microelement concentrations were higher during the first year of the trial (2016) than during the second year (2017), probably due to the washing effect produced by irrigation (Table 2). The highest concentrations were observed for DRS drainage. However, no significant differences were observed for carbonates and bicarbonates. Ammoniacal nitrogen, manganese, iron and copper presented higher values during 2017 (Table 2). Macro- and micronutrient analyses were only performed with DRS because it was the study sediment; Pt is already commercially consolidated, and the Pt-DRS values were predictably lower than the DRS values.

**Table 2:** Drainage macro and microelements analysis. Sediments 100% (DRS).

	2016	2017
**Element (mg L**^**−1**^**)**		
Sodium (Na^+^)	117	105
Potassium (K^+^)	310	139
Calcium (Ca^2+^)	198	142
Magnesium (Mg^2+^)	49.4	35.5
Boron (B^3+^)	0.791	0.636
Chlorides (Cl^−^)	214	180
Sulfates (SO_4_^2−^)	310	259
Carbonates (CO_3_^2−^)	< 5.00	< 5.00
Bicarbonates (HCO_3_^−^)	105	108
Nitrates (NO_3_^−^)	681	346
Ammoniacal nitrogen (NH_4_^+^)	0.187	1.85
Phosphates (H_2_PO_4_^−^)	56.4	26
**Element (μg L**^**−1**^**)**		
Manganese (Mn)	103	180
Iron (Fe)	457	3970
Zinc (Zn)	774	435
Copper (Cu)	127	158
Molybdenum (Mo)	74.4	50.9

#### Heavy metal contents

The heavy metal contents in the DRS drainage for 2016 and 2017 were below the maximum permissible level (MPL), as shown in Table 3. In addition, the sums of the fractions analyzed were considerably lower than the MPL, which means that the results were in accordance with the maximum legal limits. Therefore, during the first trial year (total content of 0.1156), only 3.85% of the allowed threshold was reached (Σ of fractions < 3), and this value increased to 7.25% during the second trial year (total content of 0.2173). The values were in accordance with Spanish and European legal provisions^22,23^.

**Table 3:** Heavy metals contents and the sum of the fractions analysed in DRS drainage.

Element	2016	2017	MPL	Fraction	
	(μg L^−1^)			2016	2017
Arsenic (As)	ND	ND	ND	ND	ND
Cadmium (Cd)	12.1	14.4	500	0.0242	0.0288
Chromium (Cr)	< 10.00	< 10.00	500	0.02	0.02
Nickel (Ni)	24	10.2	10,000	0.0024	0.00102
Mercury (Hg)	< 0.200	12.6	100	0.002	0.126
Lead (Pb)	7.8	< 2.00	500	0.0156	0.004
Selenium (Se)	ND	ND	ND	ND	ND
Copper (Cu)	127	158	10,000	0.0127	0.0158
Zinc (Zn)	774	435	20	0.0387	0.02175
			Σ total	0.1156	0.21737
			MPL	3	3

MPL: Maximum Permissible Level (Established by Hydraulic Public Domain Regulation, approved by the Spanish Royal Decree 849/1986); ND: Not Detected.

#### Pesticide quantification

During the assay, 613 pesticide residues were analyzed. The pesticide residues detected in the DRS drainage are shown in Table 4. The full list of pesticides can be found in the *supplementary material* attached to this paper. In general, five pesticides, acetochlor, anthraquinone, flutriafol, metalaxyl and simazine, were detected in 2016, while only two were present in 2017, acenaphthylene and phenanthrene. In all cases, the concentrations were below the Spanish MPL determined by the Hydraulic Public Domain Regulation^22^. Thus, in 2016, the sum of pesticide residues accounted for 25.86% of the MPL (0.05 mg L^−1^), and this value fell to 0.11% in 2017 (Table 4). The results were also in accordance with European legal requirements^23^.

**Table 4:** SDR drainage pesticide quantification.

Pesticide residues	Detected summary (mg L^−1^)	
	2016	2017
Acetochlor	0.01200	ND
Anthraquinone	0.00014	ND
Flutriafol	0.00006	ND
Metalaxil (+ Metalaxil-M)	0.00020	ND
Simazine	0.00053	ND
Acenaftileno	ND	0.000024
Phenanthrene	ND	0.000030
Total amount	0.01293	0.000054
MPL	0.05	0.05

MPL: Maximum Permissible Level (Established by Hydraulic Public Domain Regulation, approved by the Spanish Royal Decree 849/1986); ND: Not Detected.

### Plant material characterization

#### Heavy metal contents

The heavy metal concentrations were determined in the different strawberry plant parts (root, stem, leaves and fruit) grown in Pt (as control) and in DRS (Tables 5, 6). The analyses were carried out during the two years of the experiment (2016 and 2017). The results for the studied substrates showed that for leaf, stem and root, the most abundant metals were Mn, Fe, Zn, Al, and Sr, independent of the year. Comparing the heavy metal concentrations in the leaf, stem and root, the root presented the highest values, which is logical since the root is the plant part that has direct contact with the substrate. The Zn, Cu Ni, Cr and Pb contents in the roots were above the maximal levels for food. From an alimentary perspective, since these plant parts are not used for food, heavy metal content could be considered not to be a problem. Even so, most of the metal contents were below the recommended limits for food^24,25^.

**Table 5:** Metal ion contents (mg kg^−1^) in different strawberry plant parts grown in peat (Pt) and dredge remediated sediment (DRS).

Element	ROOT 2016		ROOT 2017		STEM 2016		STEM 2017		LEAF 2016		LEAF 2017	
	Pt	DRS	Pt	DRS	Pt	DRS	Pt	DRS	Pt	DRS	Pt	DRS
Manganese (Mn)	72.4 a	1200 b	171 a	338 b	54 b	20.5 a	83.3 b	21.2 a	86.5 b	< 20.0 a	227 b	88.5 a
Iron (Fe)	325 a	4590 b	625 a	2830 b	47.2 a	64.5 a	17.7 a	60.3 b	184 a	205 a	120 a	203 b
Zinc (Zn)	90.1 a	128 a	80.5 a	104 a	43.3 a	32.8 a	39.1 a	30.4 a	26.6 a	25.3 a	22.8 a	20.1 a
Copper (Cu)	9.47 a	96.3 b	7.5 a	41.2 b	< 2.50	3.98	< 2.50	3.18	< 2.50	4.91	< 2.50	3.38
Molybdenum (Mo)	17.5 a	43.5 b	4.08 a	196 b	0.575 a	1.08 a	0.165 a	0.333 a	0.33 a	2.02 b	0.557 a	1.16 b
Lead (Pb)	0.966 a	10.1 b	2.19 a	8.56 b	0.111 a	0.239 b	< 0.100	0.124	0.30 a	0.402 a	0.281 a	0.556 a
Cadmium (Cd)	0.193 a	1.3 b	0.327 a	1.51 b	< 0.050	0.089	0.061 a	0.22 b	0.154 a	0.275 b	< 0.050	0.173
Nickel (Ni)	0.683 a	7.18 b	0.964 a	8.23 b	0.147 a	0.266 a	0.246 a	0.360 a	0.487 a	1.32 b	0.424 a	1.16 b
Chromium (Cr)	0.74 a	7.17 b	1.35 a	7.84 b	0.209 a	0.225 a	0.323 a	0.316 a	0.693 a	1.15 a	0.567 a	0.425 b
Mercury (Hg)	< 0.010 a	0.045 b	0.037 a	0.072 b	< 0.010	< 0.010	0.043 a	0.037 a	0.013 a	0.015 a	0.043 a	0.048 b
Cobalt (Co)	< 0.200 a	6.38 b	0.258 a	2.46 b	< 0.200	< 0.200	< 0.200	< 0.200	< 0.200	< 0.200	< 0.200	0.312
Antimony (Sb)	< 0.050 a	1.07 b	< 0.05 a	0.283 b	< 0.050	< 0.050	< 0.050	< 0.050	0.024 a	0.027 a	< 0.0500	< 0.050
Arsenic (As)	0.129 a	2.19 b	0.222 a	1.54 b	0.0316 a	0.113 b	0.010 a	0.093 b	0.132 a	0.627 b	0.18 a	0.382 a
Selenium (Se)	< 0.100 a	0.711 b	0.239 a	0.795 b	< 0.100	< 0.100	< 0.100	< 0.100	< 0.100	< 0.100	< 0.100	< 0.100
Aluminium (Al)	188 a	1870 b	497 a	3150 b	38.4 a	82.5 b	10.5 a	58.7 b	260 a	285 a	110 a	177 a
Beryllium (Be)	< 0.020 a	0.149 b	0.038 a	0.162 b	< 0.020	< 0.020	< 0.020	< 0.020	< 0.02	< 0.02	< 0.02	< 0.02
Tin (Sn)	< 0.100 a	0.404 b	0.265 a	2.6 b	< 0.100	< 0.100	< 0.100	1.92	0.102 a	0.077 a	2.84 a	2.02 a
Strontium (Sr)	94.9 a	263 b	151 a	169 a	78.8 a	57.7 a	142 a	117 a	48.2 a	91.5 b	128 a	146 a
Silver (Ag)	< 0.100	< 0.100	< 0.100	< 0.100	< 0.100	< 0.100	< 0.100	< 0.100	< 0.100	< 0.100	< 0.100	< 0.100
Thallium (Tl)	< 0.020 a	0.221 a	0.038 a	0.176 a	< 0.020	< 0.020	0.040 a	0.040 a	< 0.020	< 0.020	< 0.020	< 0.020
Vanadium (V)	3.42 a	40.0 a	6.71 a	19.4 a	< 0.200	< 0.200	< 0.200	< 0.200	0.309 a	0.394 a	< 0.200	0.284

Different letters within a row for each plant part and each year indicate significant differences by Tukey’s test. (ρ ≤ 0.05). (n = 3).

**Table 6:** Metal ion contents (mg kg^−1^) in strawberry fruits grown in peat (Pt) and dredge remediated sediment (DRS).

Element (mg kg^−1^)	2016		2017	
	Pt	DRS	Pt	DRS
Total Manganese (Mn)	6.02 b	3.11 a	3.78	< 2.0
Total Iron (Fe)	10.3 a	7.92 a	2.56 a	2.51 a
Total Zinc (Zn)	3.67 a	4.31 a	1.11 a	1.41 a
Total Copper (Cu)	< 0.5	0.566	< 0.5	< 0.5
Molybdenum (Mo)	0.102 a	0.255 b	< 0.02	0.090
Lead (Pb)	< 0.02	0.06	< 0.02	< 0.02
Cadmium (Cd)	< 0.02	< 0.02	< 0.02	< 0.02
Nickel (Ni)	< 0.100	< 0.100	< 0.100	< 0.100
Chromium (Cr)	< 0.100	< 0.100	< 0.100	< 0.100
Mercury (Hg)	< 0.005	< 0.005	< 0.005	< 0.005
Cobalt (Co)	< 0.05	< 0.05	< 0.05	< 0.05
Antimony (Sb)	< 0.05	< 0.05	< 0.05	< 0.05
Arsenic (As)	< 0.05	< 0.05	< 0.05	< 0.05
Selenium (Se)	< 0.05	< 0.05	< 0.05	< 0.05
Aluminum (Al)	2.95 a	29.1 b	2.95	< 0.500
Beryllium (Be)	< 0.005	< 0.005	< 0.005	< 0.005
Tin (Sn)	< 0.2	< 0.2	0.374 a	0.669 b
Strontium (Sr)	1.85 a	2.99 a	0.729 a	1.11 a
Silver (Ag)	< 0.025	< 0.025	< 0.025	< 0.025
Thallium (Tl)	< 0.005	< 0.005	0.070	< 0.005
Vanadium (V)	< 0.05	< 0.05	< 0.05	< 0.05

Different letters within a line indicate significant differences by Tukey’s test. (ρ ≤ 0.05). (n = 3).

In addition, for the strawberry fruits (Table 6), Mn, Fe, Zn, Mo, Al, Sr and Ni were the predominant metals in the two substrates, and Sn, Co, Sb, As, Be, Mn, Ag, Tl, V, Se, Al, Pb, Cd, Cr and Hg were below the detection limit. In no case did the metal contents reach the limits established by European Legislation^25–29^, by the Food and Agriculture Organization of the United Nations (FAO) and the World Health Organization (WHO) (1995 and later revisions), by the standards of the United States Environmental Protection Agency (USEPA) or by heavy metal legislation from Australia, New Zealand, Canada, and Brazil^30^.

#### Pesticide quantification

Of the 613 pesticide residues analyzed (*see additional material*), only nitrates (101 mg kg^−1^ Pt and 99.40 mg kg^−1^ DRS) and fluorene (not detected in Pt and 4.10 µg kg^−1^ in DRS) were detected in the analyzed fruits. The fluorene content in strawberry fruit represents only 8.20% of the maximum allowed value of 0.05 mg kg^−1^ for food^31^.

On the other hand, the nitrate concentrations obtained both in Pt and in DRS were much lower than that established by European Regulation (EC) nº 1881/2006 and Regulation (EU) nº 1258/2011 for the most restrictive situation (infant food for infants and young children), < 200 mg NO^3−^ kg^−1^.

## Discussion

The experiment began in May 2016, so for the following discussion, the period from May to December 2016 is considered the first year of cultivation, while that from January to July 2017 is considered the second trial year.

According to Fig. 1, during the first year, no significant differences were detected in the needs for irrigation and drainage among the three studied substrates (Pt, Pt-DRS and DRS), although during the second year, the irrigation needs for Pt were greater than those for DRS. This trend reaches significant differences beginning in the month of June (coinciding with the period of greater crop evapotranspiration), with the water applied during June and July in the Pt crop approximately 30% higher than that applied in the DRS crop. Pt-DRS showed intermediate water needs. The DRS drainage volume during the second year was greater than the Pt drainage volume, probably due to the DRS texture being heavier than that of Pt, which facilitated the appearance of channels and cracks in the sediment that increased the collected drainage volume. Regarding the drainage electrical conductivity, no significant differences were observed throughout the experiment; the pH showed similar behavior. These results agree with another study developed with the lettuce by the same research group^2^.

Based on the analysis of mineral elements in DRS drainage, an increase of 2.60% in bicarbonates, 889.31% in ammoniacal N, 74.75% in Mn, 24.41% in Cu and 768.71% in Fe were observed during the second year (2017) when compared with the values in the first cultivation year (2016). For the rest of the analyzed elements (Table 2), during the second year, the concentration decreased by between 10.26% (for sodium) and 55.16% (for potassium), while carbonates remained below the detection limit. This decrease may be due to crop absorption and irrigation washing. Sodium, chloride, potassium, sulfate, nitrate, phosphate, bicarbonate, calcium and magnesium were the elements that contributed the most salinity. Similar results were obtained by Jayasinghe et al.^32^ in the evaluation of compost as an alternative to peat use. In addition, the results agree with Wang et al.^33^ and Kuisma et al.^34^ that studied more common alternatives growing media (rice husk, perlite, reed canary grass and coconut coir) for strawberry production, confirming the potential of DRS for agriculture media.

Analysis of the data in Table 3 shows that in general, a decrease in heavy metal concentrations in DRS drainage was observed during the second cultivation year; only the Cd, Cu and Hg contents were increased, but the values were below the limits established by the Spanish regulations on surface water quality (Royal Decree 849/1986) and to those indicated by Directive 2008/105/EC. Along the same lines, Jayasinghe et al.^32^ showed that the total and removable Cu, Zn, Cr, Mn and Pb contents in different media were significantly increased when compared to peat (control), but the values always remained below the limits recommended by the United States Environmental Protection Agency^30^.

Of the 613 pesticide residues analyzed in the DRS drainage, during the experimental period, a total of 7 pesticides were detected, 5 in 2016 and another 2 in 2017. In both cases, at levels were well below the maximum limit authorized by the Hydraulic Public Domain Spanish Regulation (Royal Decree 849/1986)^22^.

The concentration of metals in plant tissues and fruits is of great interest, while some microelements, such as iron, copper, cobalt or manganese, have positive effects for humans, and high concentrations of arsenic, cadmium, lead and zinc can be dangerous to health. Therefore, the content of metals in fruits can be an indicator of the level of contamination in the soil in which they were grown^35,36^ since a proportional relationship between the increase in the extractable concentrations of Zn and Cu and the increase in their concentrations in plant tissues has been described^35^.The contents of metal ions in the different inedible parts of the strawberry (stem and leaves) were below the legal limits. Only in the root were Zn, Cu, Ni, Cr, Cd and Pb values exceeded^24,25^. However, the root is not used for human or animal food.

For the strawberry fruits, the toxic legal limits were not reached in any case. Thus, 15 of the 21 analyzed elements were below the detection limit, and the rest were below the permissible limits for human consumption^25,27–29^. In a study on the concentrations of heavy metals in broccoli tissues grown in compost, Jayasinghe et al.^32^ reached similar conclusions. The analyses were made in plant tissues with both edible and higher concentrations of Zn. These results were also observed by Smith^37^ and Fiasconaro et al.^35^, who explained that Zn is easily transferred between tissues and usually has higher concentrations in sludge than other substrates. On the other hand, copper tends to be strongly absorbed by the soil, and its absorption by plants is regulated more effectively than that of Zn^38^; thus, the Cu concentrations in the plant and fruit are generally smaller. High contents of Zn and Cu have also been described in wastewater sludge^39^. In general, copper is not considered a toxic element. Therefore, the Cu content obtained for the strawberry fruits produced with DRS was 0.566 mg kg^−1^ in 2016, and Cu was not detected in 2017. The value was considerably less than the amounts obtained by other authors for sweet orange mesocarp (0.9–3.9 mg kg^−1^)^40^ and banana peel (12 mg kg^−1^)^41^. The value obtained was 18 times lower than the maximum limit tolerated by European regulations, 10 mg kg^−1^.

In contrast, lead is considered a potential human carcinogen, with food being the main route of exposure to this metal^42^. Generally, passive Pb absorption occurs by plants, and Pb has difficulty translocating to the fruits. In this study, the Pb content in strawberry fruits was 0.06 mg kg^−1^ in 2016 and below the detection limits (< 0.020 mg kg^−1^) during the second year of the experiment. European legislation has established a Pb concentration limit lower than 0.1 mg kg^−1^^25,27^. The Pb contents in the fruits were in accordance with those obtained by Oliva et al.^36^ for bitter orange fruits.

Cadmium can accumulate in the human body and produce adverse health effects, and food intake is an important route of entry into the human body. Alimentary cadmium content is regulated by EC Regulation nº 1881/2006, which has established 0.050 mg kg^−1^ as the maximum value in vegetables and fruits. In this case, Cd was not detected in the fruits (< 0.02 mg kg^−1^). Nickel was also not detected in the strawberry fruits. This element is considered a health hazard due to its carcinogenic activity. Oliva et al.^36^ found Ni values ranging from 0.15–1.33 mg kg^−1^ in bitter orange mesocarp, while Markert^43^ reported a normal Ni value in a reference plant of 1.5 ppm.

Fe and Mn may be considered essential elements for plants and are part of many enzyme systems. They are found in all plants in varying amounts depending on the plant species and the analyzed plant part. The Fe content in strawberry fruits did not show significant differences between those produced with DRS and those produced with Pt (control substrate).

Fruits produced with DRS showed lower values of Mn when compared to Pt fruits. In addition, during the second year, the Mn levels were below the detection limits (< 2.00 mg kg^−1^). The rest of the studied metals (Cu, Pb, Cd, Ni, Cr, Hg, Co, Sb, As, Be, Ag, Tl, V, Se and Al) showed the same trend (Table 6). Therefore, in general, the strawberry fruits produced with DRS do not contain heavy metals, and the heavy metals that were present were below the limits established by European legislation.

Pesticide residue analysis of the strawberry fruits was also performed. As mentioned, 613 pesticide residues and other contaminants were analyzed (*see supplementary material*); only fluorene (DRS: 4.10 µg kg^−1^) and nitrates (Pt: 101 mg kg^−1^ and DRS 99.40 mg kg^−1^) were detected. The fluorene concentration is below the limit established by Regulation (EU) nº 10/2011 (0.05 mg kg^−1^). The nitrate concentration was much lower than that established by European legislation^26^. Therefore, from the point of view of the presence of pesticide residues, strawberry fruits produced with DRS are exempt and hence could be suitable for consumption in both fresh and processed form.

Based on the results obtained in this work, and based on other studies that have been carried out with different plant species and with sediments from other ports, the European Commission could consider the possibility of producing a legislative change that would allow the use of this resource (DRS, dredged remediated sediments) as a new agricultural substrate to reduce the environmental and economic problems generated by these sediments.

## Conclusions

Based on the results, can be affirmed that the dredged remediated sediment used in this study can be used as a culture substrate. Its utilization can be alone or mixed with other substrates that improve its physicochemical properties, especially to achieve a lighter texture with major porosity and higher organic carbon content. This statement is supported and confirmed since the tests showed that the content of heavy metals and pesticides in both water drainage and in strawberry fruits was under the Spanish and European legal limit.

About strawberry fruits, only seven (Mn, Fe, Zn, Mo, Al, Mn and Ni) of the twenty-two metals and two (nitrates and flueorene) of the six hundred-thirteen pesticides analyzed were detected in the fruit validating its safe consumption. In all the cases, values detected were much lower than those established by specific European legislation. The results confirm the suitability of strawberry fruits for fresh and / or processed consumption with no risk. Therefore, the potential for the use of treated sediments as a viable agricultural substrate is confirmed. Thereby, it could also be used as a substrate for the regeneration of degraded soils, recovery of quarries and/or roads/accesses among other similar uses.

However, it is necessary to continue studying the treated sediment behavior, alone and/or mixed, quantitatively and qualitatively, with other substrates such as coconut fiber, biochar, perlite, among others, to improve its characteristics and to conclusively confirm the feasibility of safe use of the sediment.

The debate on the need to make substantial changes to European and Spanish legislation that allows the use of remediated marine sediment must be initiated aiming of minimizing this necessary environmental impact for the economic activity of seaports around the world.

## References

[1] Deshmukh A, Dayioglu A, Aydilek A (2020). Geoenvironmental behavior of lime-treated marine sediments. Mar. Georesour. Geotechnol..

[2] Tozzi F (2019). Remediated marine sediment as growing medium for lettuce production: assessment of agronomic performance and food safety in a pilot experiment. J. Sci. Food Agric..

[3] Buceta JL (2015). Nuevo marco para la caracterización y clasificación del material dragado en España. Ribagua.

[4] Landaeta, C. J. *Potenciales impactos ambientales generados por el dragado y la descarga del material dragado*. *Instituto Nacional de Canalizaciones. Dirección de Proyectos e Investigación* https://servicio.bc.uc.edu.ve/ingenieria/revista/a5n2/5-2-3.pdf (1995).

[5] Eggleton J, Thomas KV (2004). A review of factors affecting the release and bioavailability of contaminants during sediment disturbance events. Environ. Int..

[6] Sonneveld, C., Voogt, W., Sonneveld, C. & Voogt, W. Plant Nutrition in Future Greenhouse Production. in *Plant Nutrition of Greenhouse Crops* 393–403 (Springer Netherlands, 2009). 10.1007/978-90-481-2532-6_17.

[7] Raviv M (2013). Composts in growing media: What’s new and what’s next?. Acta Hortic..

[8] Sinclair AL (2020). Effects of distance from canal and degradation history on peat bulk density in a degraded tropical peatland. Sci. Total Environ..

[9] Tittarelli F (2009). Compost-based nursery substrates: Effect of peat substitution on organic melon seedlings. Compost Sci. Util..

[10] Shober AL (2010). Plant performance and nutrient losses during containerized bedding plant production using composted dairy manure solids as a peat substitute in substrate. Am. Soc. Hortic. Sci..

[11] Bustamante MA (2008). Composts from distillery wastes as peat substitutes for transplant production. Resour. Conserv. Recycl..

[12] Kritsotakis IK, Kabourakis EM (2011). Grape vine waste and giant reed biomass composts as peat and mineral fertilizer substitutes for producing organic tomato transplants. J. Crop Improv..

[13] Mattei P (2017). Use of phytoremediated sediments dredged in maritime port as plant nursery growing media. J. Environ. Manag..

[14] Tripathi, S. *et al.* Phytoremediation of organic pollutants: Current status and future directions. in *Abatement of Environmental Pollutants: Trends and Strategies* 81–105 (Elsevier, 2019). 10.1016/B978-0-12-818095-2.00004-7.

[15] Fayiga AO, Ma LQ, Cao X, Rathinasabapathi B (2004). Effects of heavy metals on growth and arsenic accumulation in the arsenic hyperaccumulator Pteris vittata L. Environ. Pollut..

[16] Barbara De Lucia, Giuseppe Cristiano, Lorenzo Vecchietti, Elvira Rea & Giovanni Russo. Nursery Growing Media: Agronomic and Environmental Quality Assessment of Sewage Sludge-Based Compost. *Appl. Environ. Soil Sci.* (2013).

[17] Brito LM, Reis M, Mourão I, Coutinho J (2015). Use of acacia waste compost as an alternative component for horticultural substrates. Commun. Soil Sci. Plant Anal..

[18] Melgarejo P (2017). Effect of a new remediated substrate on fruit quality and bioactive compounds in two strawberry cultivars effect of a new remediated substrate on fruit quality and bioactive compounds in two strawberry cultivars. J. Food Nutr. Res..

[19] International Organization for Standardization (ISO). *ISO 11348–3:2007/Amd 1:2018 - Water quality — Determination of the inhibitory effect of water samples on the light emission of Vibrio fischeri (Luminescent bacteria test) — Part 3: Method using freeze-dried bacteria — Amendment 1*. https://www.iso.org/standard/73342.html (2018).

[20] Ministerio de la Presidencia. *Real Decreto 865/2010, de 2 de julio, sobre sustratos de cultivo.* https://www.boe.es/buscar/doc.php?id=BOE-A-2010-11153 (2010).

[21] Scott Stieg, Bradford Fisher, Owen Mather & Theresa Wright. 4500-NH 3 NITROGEN (AMMONIA). in *Standard Methods For the Examination of Water and Wastewater* (ed. Joint Task Group) (2017).

[22] Ministerio de Obras Públicas y Urbanismo. *Real Decreto 849/1986, de 11 de abril, por el que se aprueba el Reglamento del Dominio Público Hidráulico, que desarrolla los títulos preliminar I, IV, V, VI y VII de la Ley 29/1985, de 2 de agosto, de Aguas.* https://www.boe.es/buscar/act.php?lang=en&id=BOE-A-1986-10638&tn=&p= (2018).

[23] European Parliament. *Directive 2008/105/EC of the European Parliament and of the Council of 16 December 2008 on environmental quality standards in the field of water policy, amending and subsequently repealing Council Directives 82/176/EEC, 83/513/EEC, 84/156/EEC, 84/491/EEC, 86/280/EEC and amending Directive 2000/60/EC of the European Parliament and of the Council*. https://eur-lex.europa.eu/legal-content/EN/TXT/?uri=CELEX:02008L0105-20130913 (2013).

[24] Commision, C. A. *General Standard for contaminants and toxins in food and feed (CODEX STAN 193–1995)*. (2016).

[25] Commission Regulation (EC). *No 1881/2006 of 19 December 2006 setting maximum levels for certain contaminants in foodstuffs*. https://eur-lex.europa.eu/legal-content/EN/ALL/?uri=CELEX%3A32006R1881 (2006).

[26] Commission Regulation (EU). *N*^*o*^* 1258/2011 of 2 December 2011 amending Regulation (EC) No 1881/2006 as regards maximum levels for nitrates in foodstuffs*. https://eur-lex.europa.eu/legal-content/EN/ALL/?uri=CELEX%3A32011R1258 (2011).

[27] Commission Regulation (EU). *N*^*o*^* 2015/1005 of 25 June 2015 amending Regulation (EC) No 1881/2006 as regards maximum levels of lead in certain foodstuffs*. https://op.europa.eu/en/publication-detail/-/publication/f7405b97-1bc7-11e5-a342-01aa75ed71a1/language-en/format-PDF/source-123143860 (2015).

[28] Commission Regulation (EU). *N*^*o*^* 2015/1006 of 25 June 2015 amending Regulation (EC) No 1881/2006 as regards maximum levels of inorganic arsenic in foodstuffs (Text with EEA relevance)*. https://op.europa.eu/en/publication-detail/-/publication/4ea62ae9-1bc8-11e5-a342-01aa75ed71a1/language-en/format-PDF/source-123143938 (2015).

[29] Commission Regulation (EU). *N*^*o*^* 488/2014 of 12 May 2014 amending Regulation (EC) No 1881/2006 as regards maximum levels of cadmium in foodstuffs*. (2014).

[30] United Stated Environmental Protection Agency. *Biosolids Generation, Use, and Disposal in the United States*. (1990).

[31] Commision Regulation (EU). *No 10/2011 of 14 January 2011 on plastic materials and articles intended to come into contact with food*. https://eur-lex.europa.eu/LexUriServ/LexUriServ.do?uri=OJ:L:2011:012:0001:0089:EN:PDF (2011).

[32] Jayasinghe GY, Arachchi IDL, Tokashiki Y (2010). Evaluation of containerized substrates developed from cattle manure compost and synthetic aggregates for ornamental plant production as a peat alternative. Resour. Conserv. Recycl..

[33] Wang D, Gabriel MZ, Legard D, Sjulin T (2016). Characteristics of growing media mixes and application for open-field production of strawberry (*Fragaria ananassa*). Sci. Hortic. (Amsterdam).

[34] Kuisma E, Palonen P, Yli-Halla M (2014). Reed canary grass straw as a substrate in soilless cultivation of strawberry. Sci. Hortic. (Amsterdam).

[35] Fiasconaro ML, Antolín MC, Lovato ME, Gervasio S, Martín CA (2015). Study of fat compost from dairy industry wastewater as a new substrate for pepper (*Capsicum annuum* L.) crop. Sci. Hortic. (Amsterdam).

[36] Oliva SR, Valdés B, Mingorance MD (2008). Evaluation of some pollutant levels in bitter orange trees: implications for human health. Food Chem. Toxicol..

[37] Smith SR (2009). A critical review of the bioavailability and impacts of heavy metals in municipal solid waste composts compared to sewage sludge. Environ. Int..

[38] Kabata-Pendias, A. Soil-plant transfer of trace elements - An environmental issue. in *Geoderma* vol. 122 143–149 (Elsevier, 2004).

[39] Antolín MC, Pascual I, García C, Polo A, Sánchez-Díaz M (2005). Growth, yield and solute content of barley in soils treated with sewage sludge under semiarid Mediterranean conditions. F. Crop. Res..

[40] Crescimanno FG, Germanã MA, Melati MR, Orecchio S, Vitale MC (2000). Secondary stress several edible citrus cultivars caused by heavy metal air pollution. Aerobiologia (Bologna)..

[41] Selema MD, Farago ME (1996). Trace element concentrations in the fruit peels and trunks of Musa paradisiaca. Phytochemistry.

[42] Capdevila F, Nadal M, Schuhmacher M, Domingo JL (2003). Intake of lead and cadmium from edible vegetables cultivated in Tarragona Province Spain. Trace Elem. Electrolytes.

[43] Markert BA (1996). Instrumental Element and Multi-element Analysis of Plant Samples: Methods and Applications.

